# Causes of hepatic capsular retraction: a pictorial essay

**DOI:** 10.1007/s13244-016-0520-7

**Published:** 2016-09-29

**Authors:** Gary Xia Vern Tan, Rhian Miranda, Tom Sutherland

**Affiliations:** 1 Department of Medical Imaging, St Vincent’s Hospital Melbourne, 41, Victoria Parade, Fitzroy, Victoria 3065 Australia; 2 Auckland City Hospital, Auckland, New Zealand

**Keywords:** Liver, Capsular retraction, Computed tomography, Liver abnormalities, Pseudo-retraction

## Abstract

**Abstract:**

Hepatic capsular retraction refers to the loss of the normal convex hepatic contour, with the formation of an area of flattening or concavity. This can result from myriad causes, including intrinsic hepatic conditions such as cirrhosis, biliary obstruction, benign tumours, malignancy and infections, as well as extrahepatic causes such as trauma. This article aims to provide familiarity with this wide spectrum of conditions, including mimics of hepatic capsular retraction, by highlighting the anatomic, pathologic and imaging features that help distinguish these entities from one another.

***Teaching Points*:**

*• Hepatic capsular retraction can occur due to various intrinsic or extrinsic hepatic causes.*

*• Hepatic capsular retraction is observed in both benign and malignant conditions.*

*• Recognising associated imaging features can help elicit causes of hepatic capsular retraction.*

## Introduction

Hepatic capsular distortion can occur in a variety of entities, both intrahepatic and extrahepatic, including cirrhosis, benign neoplasia, malignant distortion and hepatic trauma. This article aims to provide familiarity with this wide spectrum of conditions, as well as conditions that produce an appearance mimicking hepatic capsular retraction on imaging, by highlighting the anatomic, pathologic and imaging features that help distinguish these entities from one another Table 1.

**Table 1:** Causes of hepatic capsular retraction

Intrahepatic causes	
Cirrhosis	
Hepatocellular carcinoma	
Cholangiocarcinoma	
Hepatic metastasis	
Haemangioma	
Hepatic inflammatory pseudotumour	
Biliary obstruction*	
Infections	
Fulminant hepatic necrosis	
Extrahepatic causes	
Diaphragm*	
Trauma	
Treated tumours	
Pseudomyxoma peritonei*	
Pseudolipoma of Glisson’s capsule*	

*Mimics hepatic capsular retraction

## Hepatic capsule anatomy

**Fig. 1:**
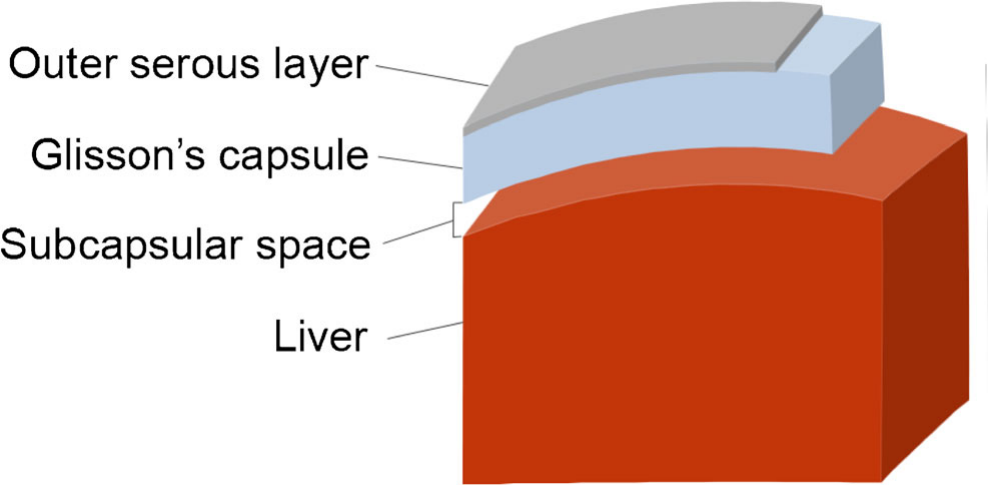
Schematic representation of the anatomy of the hepatic capsule. Note that the subcapsular space is a potential space

The hepatic capsule is divided into two adherent layers, an outer serous layer and an inner fibrous layer. The outer serous layer is derived from the peritoneum and covers most of the liver surface, but not the bare area of the liver, which is near the diaphragm, the porta hepatis, or the area where the gallbladder is attached to the liver. The inner layer is a thick fibrous layer which consists of type III collagen and extends to the stroma of endothelial sinusoids. Also known as Glisson’s capsule, this fibrous layer covers the entire surface of the liver, unlike the outer serous layer [1]. Superficial to the liver parenchyma and deep to Glisson’s capsule is a potential space, the subcapsular space, which has the potential to hold fluid, blood or cells (Fig. 1).

Neither the serous nor fibrous liver capsule is visible in normal conditions under CT or MR imaging and maintains a strict adherence to the liver parenchyma. It does become visible, however, in a number of entities both benign and malignant [2].

Hepatic capsular retraction is defined as loss of the normal convex hepatic contour, an area of focal flattening or concavity. This can be seen secondary to intrinsic subcapsular liver lesions, which cause true capsular retraction by pulling the liver edge away from the hepatic capsule. Alternatively, the loss of normal convex hepatic contour can also be seen in a number of normal variations such as accessory hepatic fissure and invagination of the liver by the diaphragm. Pseudoretraction can also be demonstrated when fluid, blood and both benign and malignant deposits are seen in the subcapsular space. These typically have a lentiform appearance and can give the liver edge a similar concave appearance.

### Intrahepatic causes

#### Cirrhosis

**Fig. 2:**
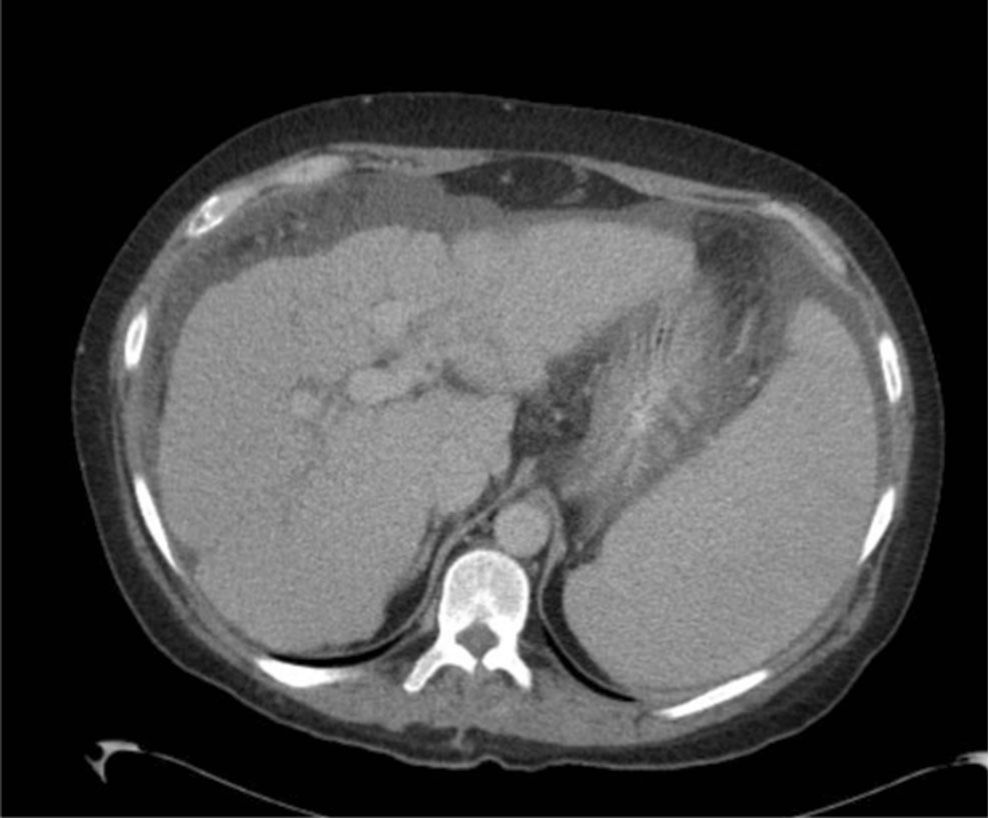
Portal venous CT demonstrates an irregular nodular liver, with the capsule outlined by ascites. Note splenomegaly due to portal hypertension

Cirrhosis occurs in the setting of diffuse hepatocyte injury, resulting in disruption of hepatic architecture with the formation of fibrous septae and regenerative nodules, both micronodular (<3 mm) and macronodular (>3 mm) [3]. Imaging findings in cirrhosis include caudate lobe hypertrophy, atrophy of other lobes and parenchymal nodularity, which is most apparent along the liver surface. This results in distortion of the liver contour, most apparent in cases of macronodular cirrhosis (Fig. 2). Micronodular cirrhosis may not be apparent on imaging examinations.

**Fig. 3:**
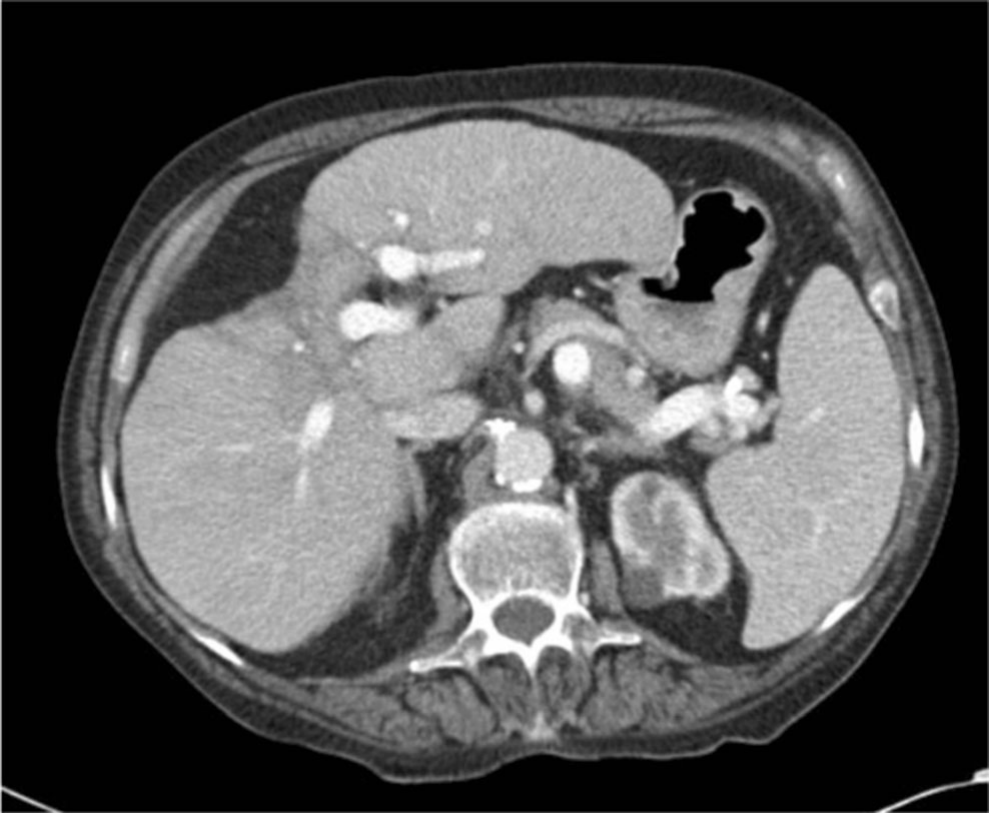
Dumbbell-shaped liver due to confluent fibrosis. This retracts the capsule overlying segment 4 with a subtle heterogeneous area of hypo-enhancement in segment 4. The remainder of the liver has a slightly nodular contour due to cirrhosis

Focal confluent fibrosis is one of the grossly demonstrable patterns of fibrosis in advanced cirrhosis, most commonly seen in alcoholic cirrhosis, where it can be mistaken for malignant neoplasia. It appears as a wedge-shaped area of low attenuation on CT with volume loss. Ninety percent of cases involve the medial segment of the left lobe and/or the anterior segment of the right lobe, with sparing of the caudate and lateral segments. Capsular retraction is also demonstrated in 90 % of cases, and has been found to increase with the evolution of focal confluent fibrosis [4, 5] (Fig. 3). Eighty percent of the time it is isodense to liver parenchyma on the portal venous phase and shows delayed-phase hyper-enhancement like other fibrotic liver lesions. On MRI, it is typically hypointense to liver parenchyma on T1-weighted images and hyperintense to the liver on T2-weighted images, due to oedema and compressed remnants of the portal triads. Typically, the lesions are hypointense to the liver on the immediate post-contrast T1 sequences, with the more fibrotic areas becoming isointense to the liver on the later dynamic phases. These areas are typically hypo-enhancing in the hepatobiliary phase due to the paucity of hepatocytes. The location, shape and enhancement characteristics help differentiate confluent hepatic fibrosis from other differential diagnoses, which include cholangiocarcinoma, hepatocellular carcinoma, biphenotypic hepatocholangiocarcinoma, hepatic infarction and epithelioid haemangioendothelioma.

#### Conventional hepatocellular carcinoma (HCC)

**Fig. 4:**
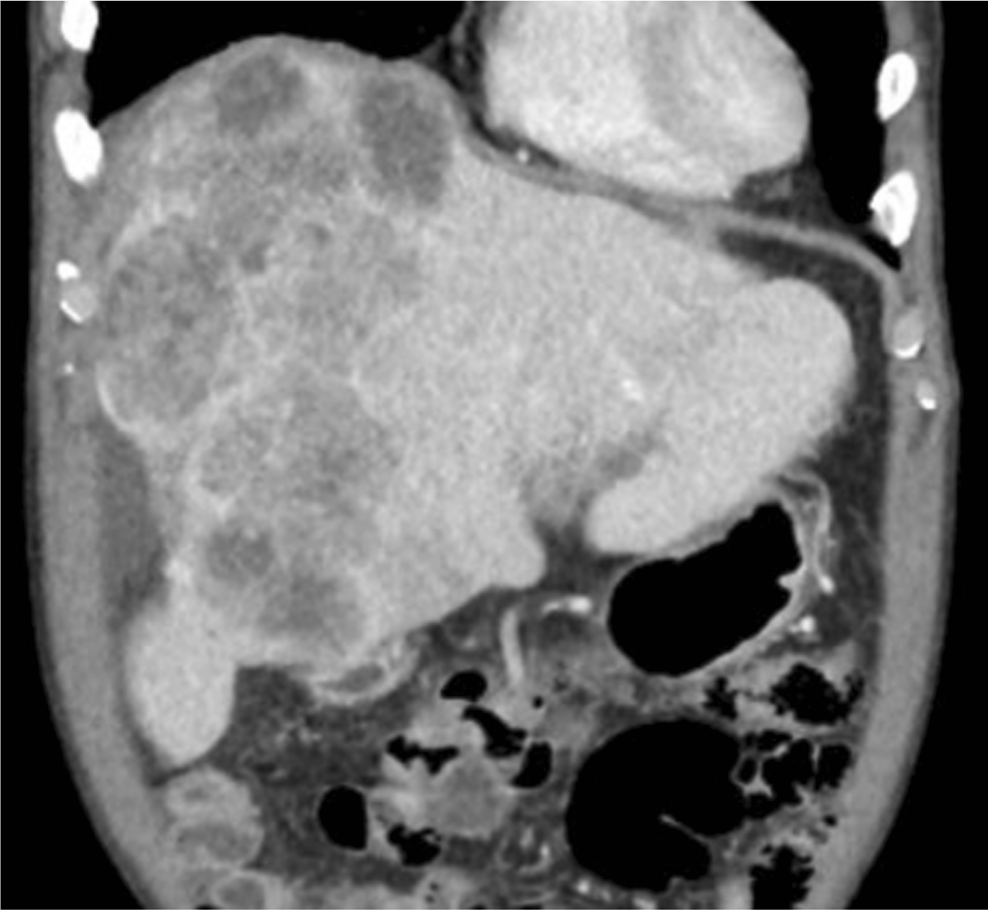
Coronal portal venous phase CT demonstrates washout in the heterogeneous HCC filling much of the right lobe of liver. Capsular retraction is present laterally with ascites filling the void

Hepatocellular carcinoma (HCC) is one of the leading causes of cancer-related deaths worldwide, being the fifth most common cancer in men and eighth most common cancer in women overall. Conventional HCC rarely contains fibrous tissue, and as such, hepatic capsular retraction is very uncommon in conventional HCC, although it can occur in the rarer subtypes that contain a greater volume of fibrosis such as sclerotic and mixed cholangio-HCC [6]. Capsular retraction in the setting of HCC is most frequently encountered after treatment, either local or systemic [7]. Transcatheter arterial chemo/radio-embolisation of hepatocellular carcinoma is a widely used method in the treatment of HCC. Chemoembolisation disrupts the arterial supply to HCC, thus depriving it of its source of nutrients, leading to ischemic necrosis within the tumour [8]. This leads to atrophy of the surrounding liver parenchyma and can cause capsular retraction of the liver (Fig. 4).

#### Cholangiocarcinoma

**Fig. 5:**
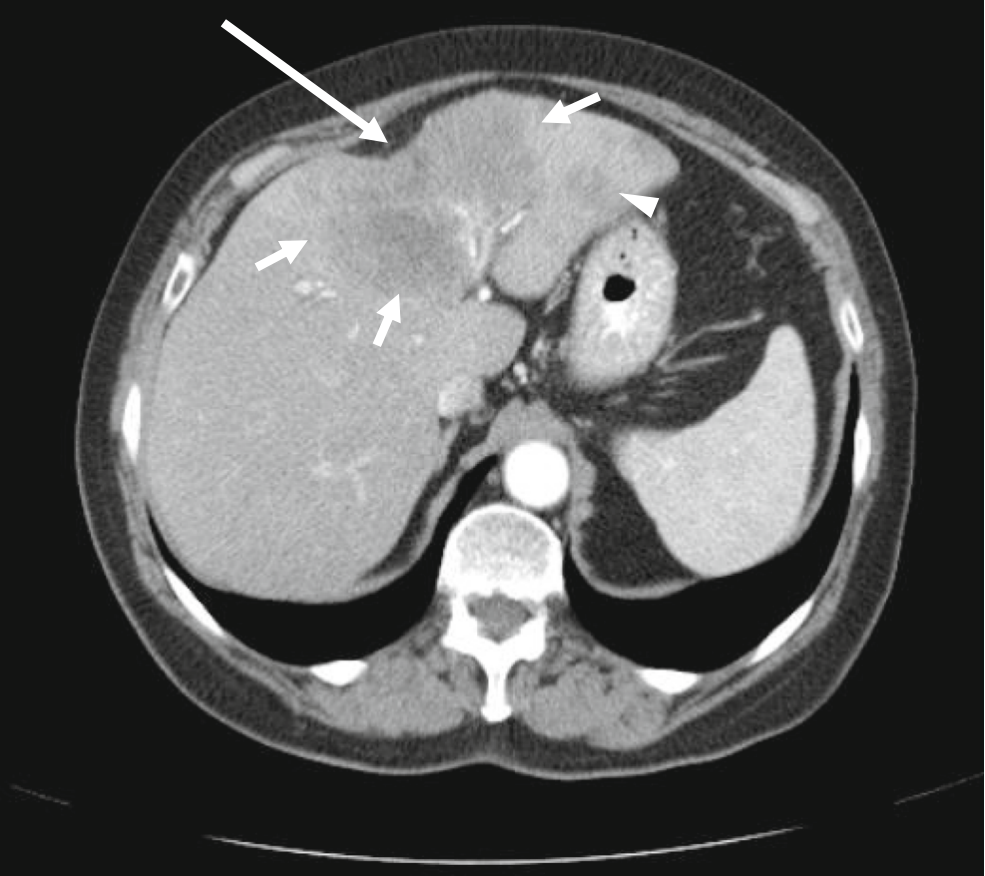
Portal venous CT demonstrates capsular retraction (*long arrow*) secondary to an infiltrating, hypo-enhancing cholangiocarcinoma (*short arrows*). Note a satellite lesion in segment 2 (*arrowhead*)

Cholangiocarcinoma is a malignant tumour arising from the biliary tree, associated with poor prognosis and high morbidity [9]. It is the second most common primary malignant hepatic tumour after hepatocellular carcinoma. Intrahepatic cholangiocarcinomas can be classified into three types on the basis of their gross morphologic features, with each type having its own characteristic imaging findings. These three morphological classifications are mass-forming, periductal-infiltrating and intraductal-growth types [10]. The mass-forming type of cholangiocarcinoma is the commonest and has the most recognised association with hepatic capsular retraction [11]. Mass-forming cholangiocarcinoma presents as an irregular, markedly low-attenuation mass with minimal irregular peripheral enhancement and focal dilatation of intrahepatic ducts around the tumour. The central part of the tumour does not enhance on arterial and portal venous phases, and there may be prolonged enhancement on the delayed phase. Capsular retraction occurs commonly in these tumours due to their variable but typically marked central fibrosis (Fig. 5). Features such as vascular encasement without grossly visible tumour thrombus, as well as the presence of satellite nodules, can help distinguish cholangiocarcinoma from other malignancies. Clinically, cholangiocarcinoma typically occurs in patients with pre-existing bile duct diseases such as biliary lithiasis, clonorchiasis, recurrent pyogenic cholangitis or primary sclerosing cholangitis, although patients with chronic liver disease are also at increased risk.

#### Hepatic metastases

**Fig. 6:**
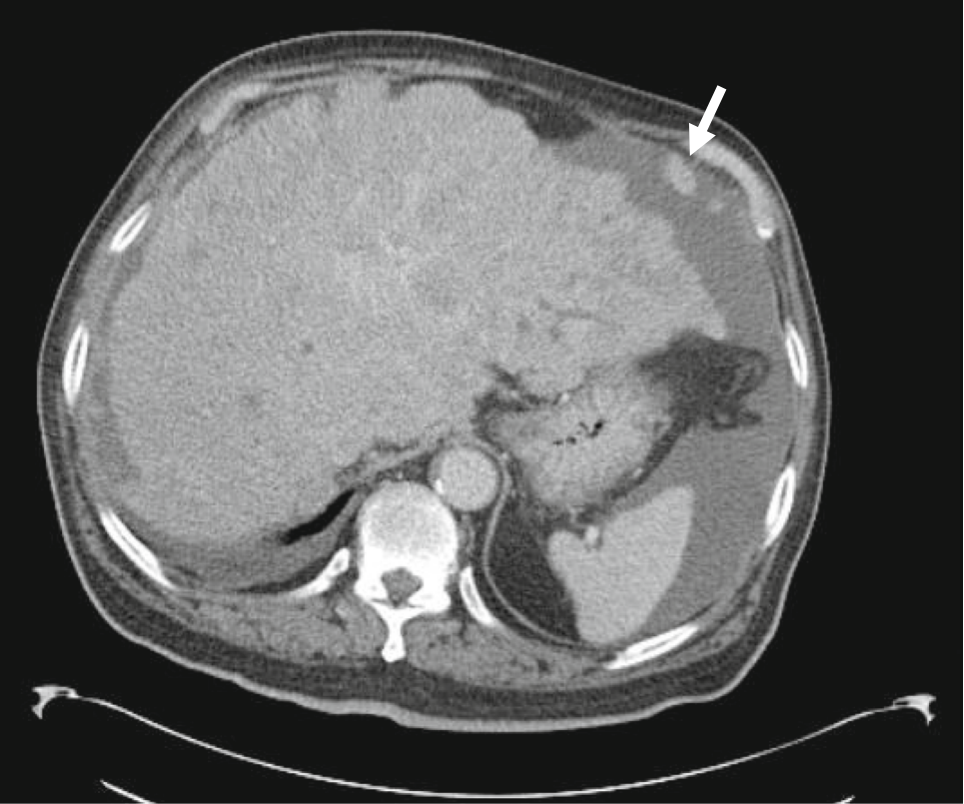
Portal venous phase CT demonstrates an enlarged liver with a markedly irregular contour due to metastatic melanoma. The patient has ascites and peritoneal deposits (*arrow*), and clinically was heavily jaundiced, with gross hepatic dysfunction. Note normal-sized spleen

**Fig. 7 & 8:**
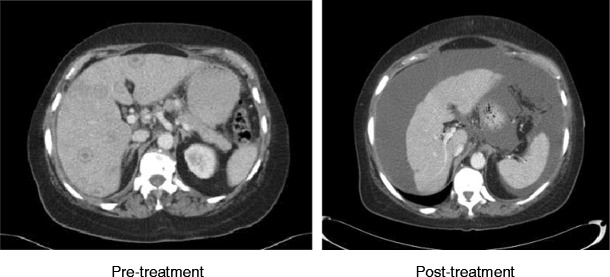
Post-treatment CT shows a small liver with nodular contour on a background of ascites but with a normal-sized spleen. The patient had completed chemotherapy for metastatic breast carcinoma with multiple hepatic metastases. The sites of retraction correspond to sites of tumour on the pre-treatment CT

Hepatic capsular retraction has a well-established association with hepatic metastases, and in the past has erroneously been claimed to be a specific sign for hepatic malignancy [12]. Metastases may cause capsular retraction in their own right, particularly with relatively fibrous primary tumours such as lung, breast and colon carcinomas and carcinoids [12, 13] (Fig. 6). Breast cancer metastases in particular have been shown to be a cause of massive capsular retraction, with both increases or decreases in the size of metastases, independent of tumour factors and chemotherapy regime [14]. These areas of multifocal capsular retraction in breast cancer metastases have thus also been classified as pseudocirrhosis of the liver [15–17]. Capsular retraction, however, is more commonly seen in metastases after they have undergone treatment with chemotherapy, radiotherapy or percutaneous ablation, as the subsequent necrosis can result in fibrous scarring (Figs. 7 and 8). On imaging, hepatic metastases vary widely in appearance, but are often multiple, spherical, and tend to appear hypo-attenuating in comparison to the remaining liver parenchyma on CT. Hypervascular metastases can be seen in endocrine tumours such as islet cell, thyroid and carcinoid. Narrowing metastases as the aetiology of capsular retraction is often based on both imaging appearance and clinical information, including a history of a primary malignancy and identification of metastatic disease elsewhere.

#### Haemangioma

**Fig. 9 & 10:**
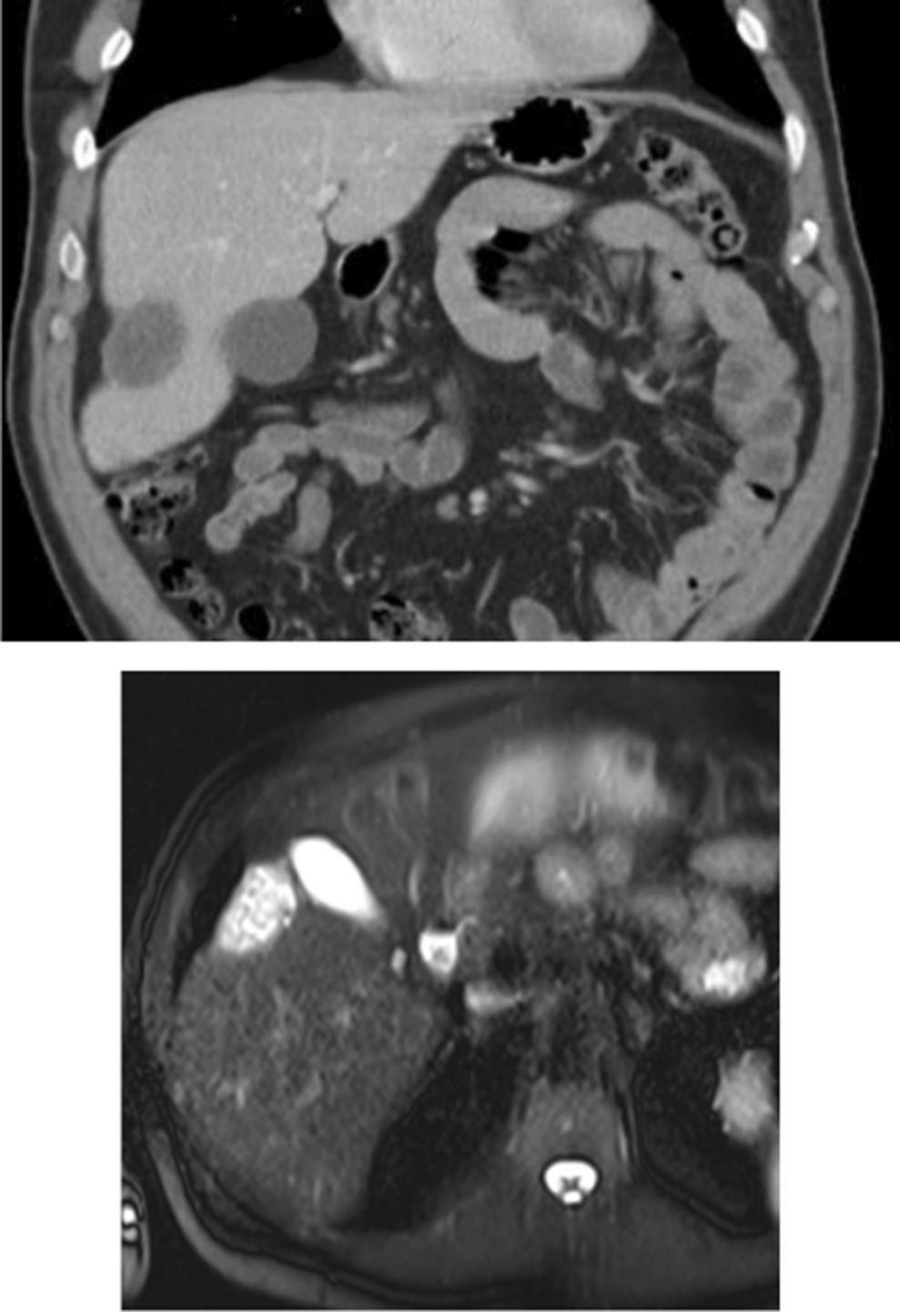
Axial CT (*upper image*) demonstrates a non-enhancing low-density solitary liver lesion with capsular retraction in a patient with newly diagnosed rectal carcinoma. The density of the lesion is slightly greater than the gallbladder. On T2-weighted MRI (*lower image*), the lesion is heterogeneous and hyperintense with capsular retraction. No enhancement was present (not shown). The lesion was resected as a suspected mucinous metastatic deposit, but histology showed a sclerosed haemangioma

Hepatic haemangiomas are the most common benign tumour of the liver, and are found in 5–20 % of the population, being the most common liver tumour overall [18, 19]. They can range in size from a few millimetres to greater than 20 cm, and are arbitrarily termed ’giant’ haemangioma when they are larger than 4 cm [20, 21]. Capsular retraction is not a typical feature, and is believed to occur in haemangiomas which undergo fibrous degeneration, although this is not always the case [22]. In the setting of cirrhosis, Brancatelli et al. showed capsular retraction in 24 % of haemangiomas [23]. In imaging with both CT and MRI, haemangiomas show typical early peripheral, nodular or globular discontinuous enhancement, with progressive centripetal enhancement to uniform filling on the venous phase. Sclerosed haemangiomas lack peripheral nodular enhancement, do not progressively fill, and may have reduced T2 signal within them. They are hypointense on the hepatobiliary phase, and will frequently have reduced apparent diffusion coefficient (ADC) values which mimic metastatic disease. Due to this atypical appearance, histological sampling is frequently required for definitive diagnosis (Figs. 9 and 10). Haemangiomas in cirrhotic livers may not demonstrate the typical radiological features, and have been shown to undergo progressive fibrosis and decrease in size, which likely accounts for variation in imaging appearance.

#### Hepatic inflammatory pseudotumour

**Fig. 11:**
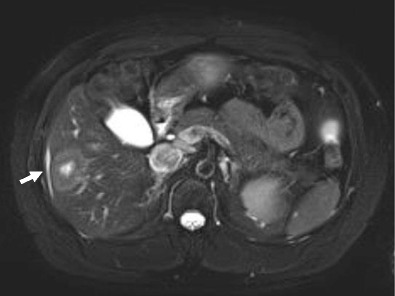
T2-weighted MRI demonstrates inflammatory pseudotumour as a target type lesion with markedly hyperintense centre and a mildly hyperintense margin in hepatic segment 5, with associated flattening of the overlying hepatic capsule

Inflammatory pseudotumour is an uncommon benign lesion within the liver, which is histologically characterised by proliferation of plasma cell histiocytes and other inflammatory cells intermingled with varying degrees of fibrosis [24]. Hepatic inflammatory pseudotumour tends to occur in young adult men, although it has been observed across all age groups [24]. These pseudotumours often pose a diagnostic dilemma, as they are difficult to differentiate from malignant tumours on imaging [25]. Several case reports have demonstrated capsular retraction associated with more peripheral hepatic inflammatory pseudotumours (Fig. 9), thus initially raising concern for an underlying malignant lesion such as cholangiocarcinoma, which was later disproved by liver biopsy [25]. Given the non-specific appearance of hepatic inflammatory pseudotumours, liver biopsy remains the gold standard for diagnosis (Fig. 11).

#### Biliary obstruction

Both benign and malignant causes of biliary obstruction can lead to lobar atrophy within the liver, with the most common causes being post-cholecystectomy stricture and cholangiocarcinoma [26]. Benign strictures causing lobar or segmental biliary obstruction result in atrophy in the lobe or segment of the liver drained by the obstructed duct [26]. Cholangiocarcinoma can also result in atrophy secondary to biliary obstruction and/or portal vein branch compromise [26]. In both scenarios, there is often hypertrophy of the contralateral hepatic lobe. At the junction between the atrophied and hypertrophied lobe or segment, there is usually a change in hepatic contour, or ‘step’, which results in distortion of the overlying hepatic capsule that can mimic hepatic capsular retraction (Fig. 10). The pattern of atrophy is also influenced by the biliary ductal anatomy, which has multiple normal variants.

#### Infections

Hepatic abscesses, particularly those located peripherally, can cause hepatic capsular retraction as they regress, even without percutaneous drainage [27]. This relates to the associated scarring and fibrosis during the recovery phase [27]. Similarly, peripherally located hepatic hydatid cysts which have ruptured can also cause capsular retraction.

**Fig. 12:**
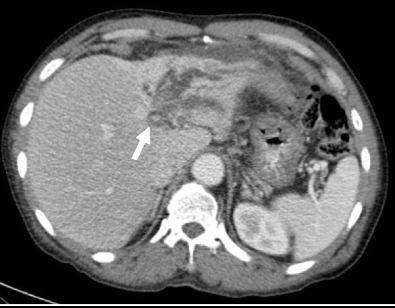
Atrophy in the left lateral segment with distended bile ducts secondary to clonorchiasis infection. Note the presence of a central hyperdense intrahepatic ductal stone (*arrow*)

Clonorchiasis is a trematodiasis caused by chronic infestation of *Clonorchis sinensis*, which can lead to recurrent pyogenic cholangitis, biliary strictures and cholangiocarcinoma. It is acquired after ingestion of raw flesh and freshwater fish in endemic areas. At imaging, clonorchiasis is best diagnosed by cholangiography, either directly (percutaneous transhepatic cholangiography or endoscopic retrograde cholangiopancreatography [ERCP]) or indirectly via magnetic resonance cholangiopancreatography (MRCP) or CT intravenous cholangiography. In particular, radiologists should suspect clonorchiasis when there is evidence of biliary obstruction with dilatation of the peripheral intrahepatic ducts, without dilatation of the extrahepatic bile duct [28]. This biliary obstruction then results in atrophy of that hepatic lobe and associated distortion of the overlying hepatic capsule. The best diagnostic clues for clonorchiasis include hepatic atrophy and the presence of hyperdense intrahepatic calculi, with a predilection for the left lobe of the liver. Rarely, clonorchis sinensis can present as filling defects, which can be a few millimetres to 10 mm long, with intrahepatic duct dilatation (Fig. 12).

#### Fulminant hepatic necrosis

**Fig. 13 & 14:**
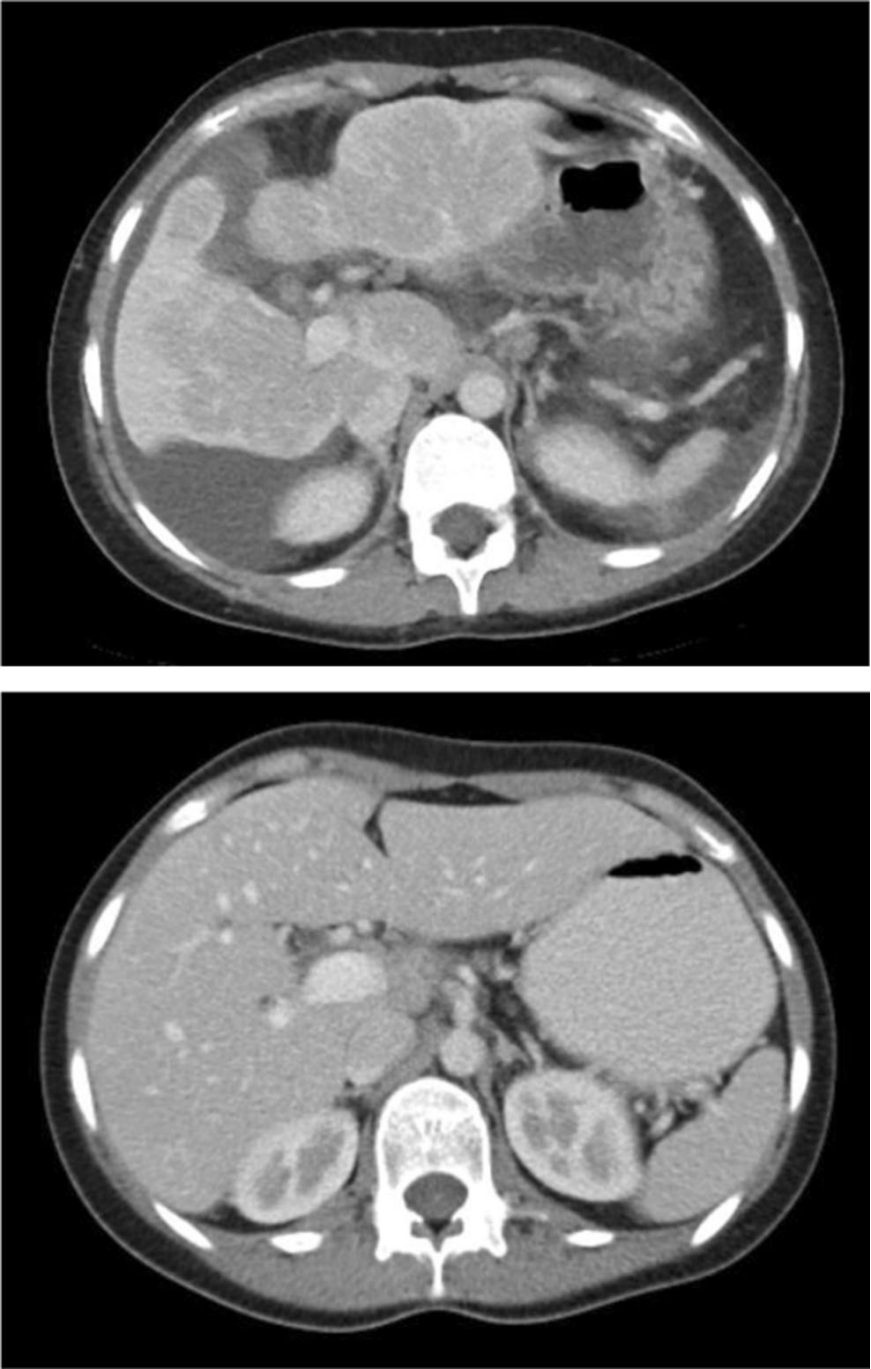
Portal venous phase CT shows multiple hypo-enhancing pseudomasses representing areas of hepatic necrosis with background of ascites. This is biopsy-proven fulminant hepatic necrosis. The necrosis-induced fibrosis and atrophy cause hepatic capsular retraction, and there has been substantial volume loss compared with a CT performed 7 months earlier (*bottom image*)

Fulminant hepatic failure is clinically characterised by acute severe impairment of hepatic function, causing hepatic encephalopathy within 8 weeks of jaundice [29]. In developing countries, viral hepatitis is the leading cause of acute liver failure, whereas in developed countries, drug-induced liver injury, such as paracetamol overdose, is the primary cause [29]. In fulminant hepatic failure, there is massive hepatocyte necrosis, which appears as areas of hypodensity on CT imaging [30]. If the patient survives, these large areas of necrosis involute due to scarring and fibrosis, resulting in hepatic volume loss and retraction of the overlying hepatic capsule [31] (Figs. 13 and 14). The formation of regenerating nodules also contributes to the irregularity of the liver margin. These areas of regenerating nodules are hyper-enhancing on contrast-enhanced imaging compared with the devascularised non-enhancing necrotic liver [31].

### Extrahepatic causes

#### Diaphragm

**Fig. 15:**
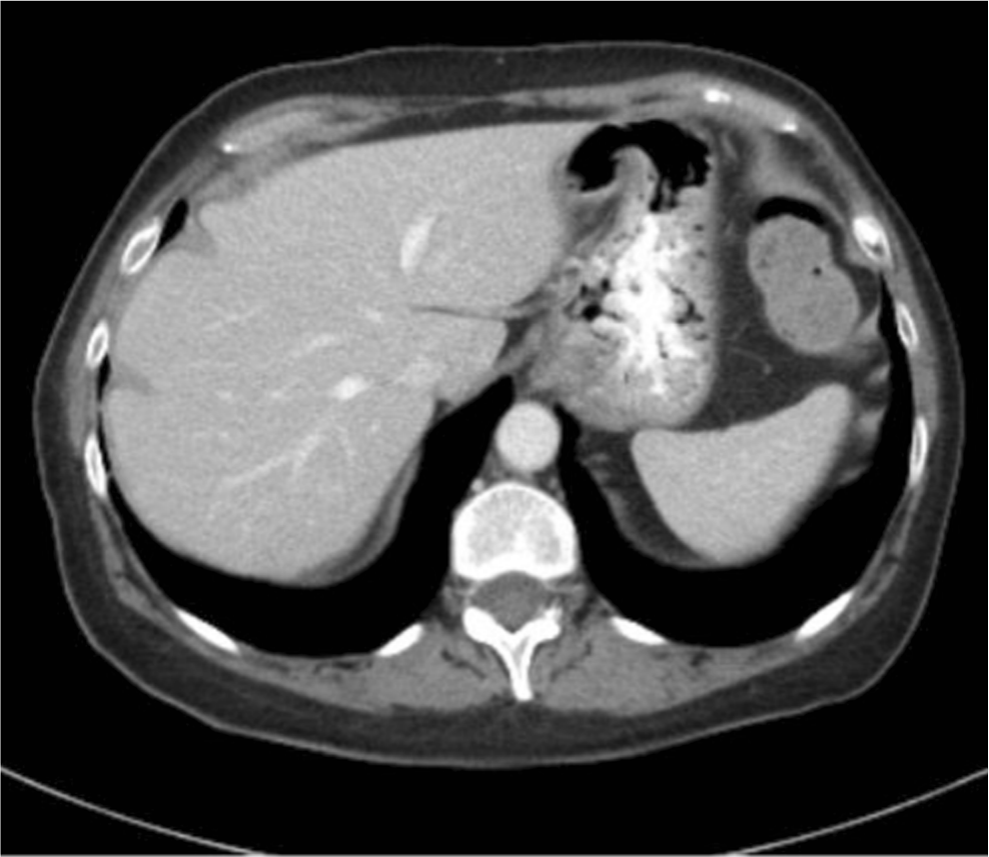
Two hypo-enhancing extrinsic indentation over the lateral liver. These appear linear on sequential slices (not shown) and each can be followed to join an overlying rib

Diaphragmatic slips can produce extrinsic indentations of the liver which may mimic hepatic capsular retraction. The key to diagnosis is to note that these are typically obliquely oriented across the anatomical right lobe of the liver, are multiple in number, and with each band of indentation leading to a rib. The ribs are located at the inferior aspect of the indentation (Fig. 15).

#### Trauma

**Fig. 16 & 17:**
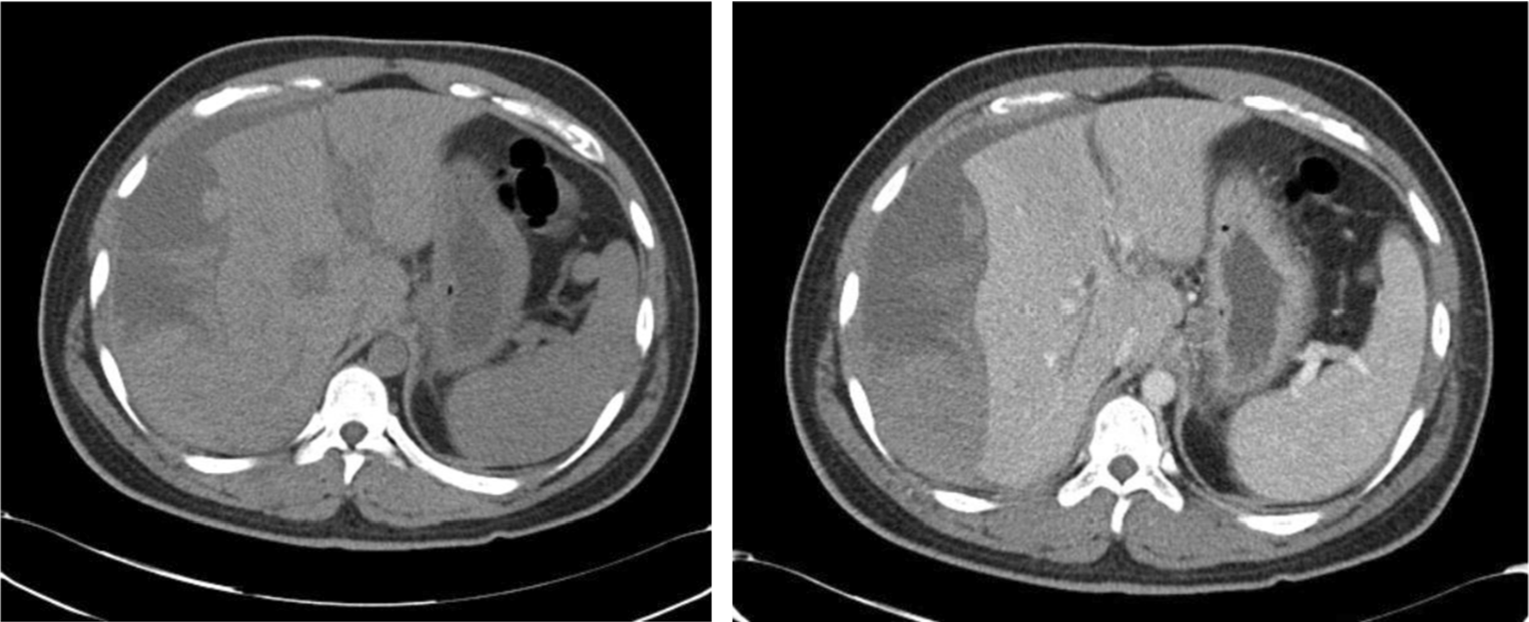
Non-contrast CT (*left*) showing a heterogenous subcapsular haematoma indenting the liver post liver biopsy. There is no enhancement or active bleeding on portal venous phase (*right*). Note the heterogeneity of the lesion with serum being anti-dependent, and more solid components of the haematoma being dependently positioned

**Fig. 18:**
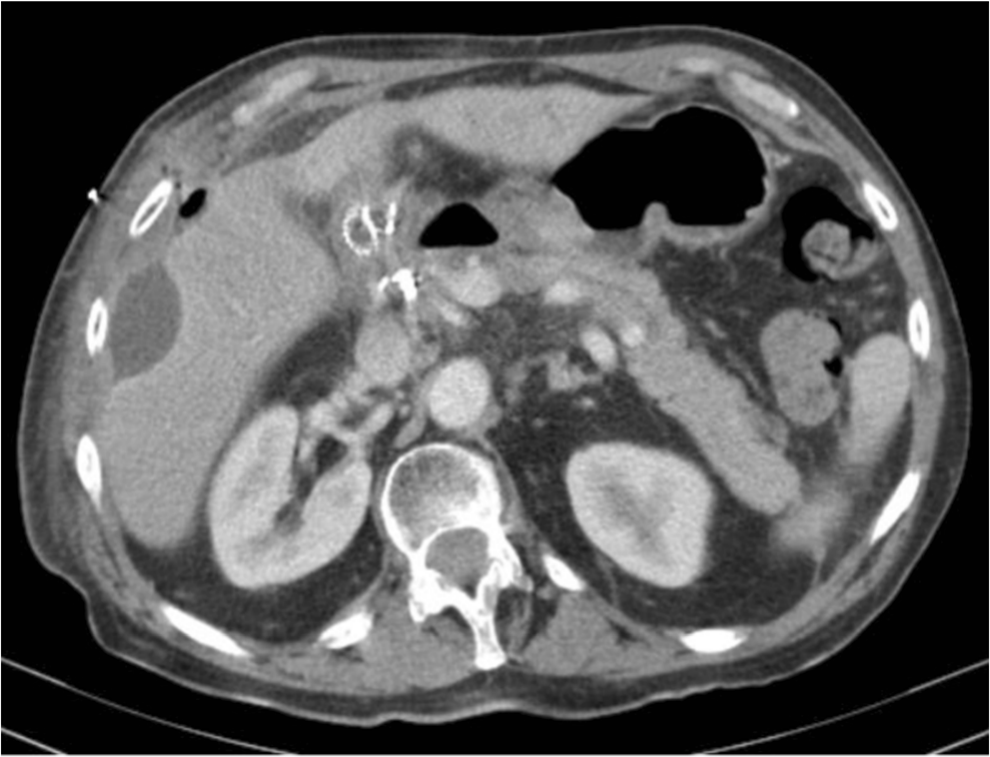
Well-defined fluid attenuation subcapsular biloma indents the liver following percutaneous transhepatic biliary intervention

**Fig. 19:**
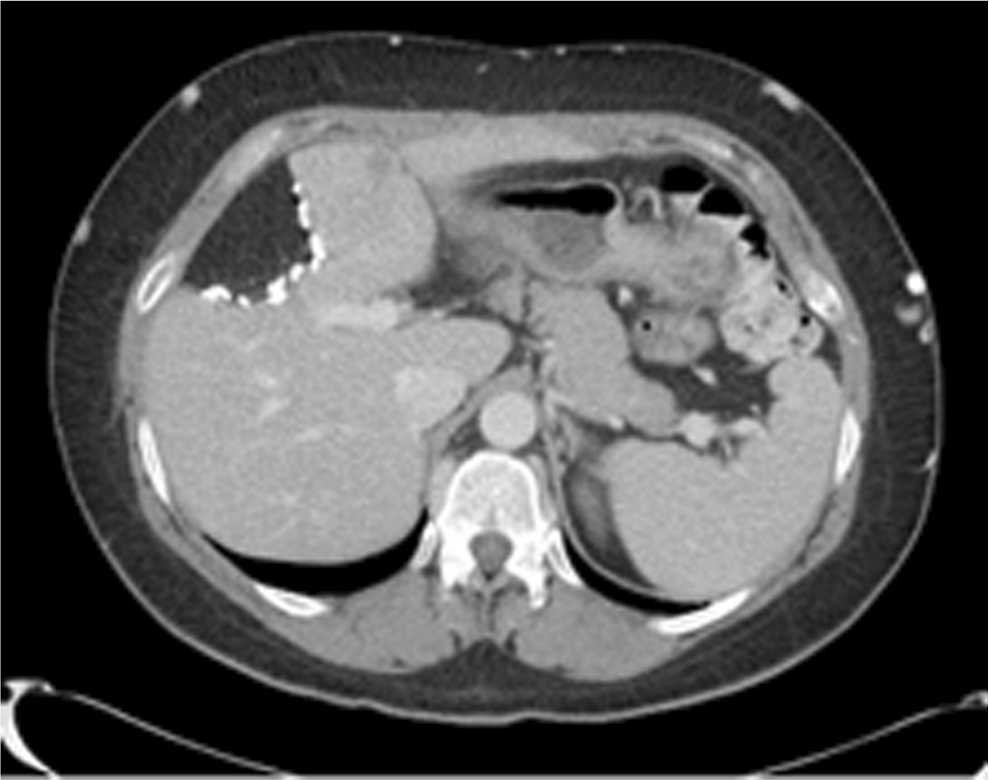
Surgical wedge resection with multiple surgical clips along the cut liver surface, with the defect being filled with fat

Capsular retraction can be seen following liver trauma, both penetrating and non-penetrating insults. The liver is the most frequently injured organ in abdominal blunt trauma [32]. CT features of both blunt and penetrating hepatic trauma include hepatic lacerations, subcapsular and parenchymal haematomas, active haemorrhage and juxtahepatic venous injuries. Capsular distortion can be seen in the acute phase of trauma, with subcapsular haematomas presenting at CT as an elliptic collection of blood trapped between the liver parenchyma and hepatic capsule. Parenchymal haematomas are seen at CT as focal low-attenuation areas with poorly defined irregular margins within the liver parenchyma on contrast-enhanced CT (Figs. 16 and 17). Hepatic lacerations may also cause bile leakage with biloma formation, which may result in necrosis of the surrounding hepatic parenchyma (Fig. 18). At follow-up, a rare complication of trauma includes abscess formation, both hepatic and perihepatic, and has been reported in up to 4 % of blunt liver trauma not undergoing surgical management [33, 34]. Fibrosis as a result of healing of the hepatic and perihepatic abscess can lead to capsular distortion (Fig. 19).

Iatrogenic hepatic trauma can also result in hepatic capsular retraction due to the fibrosis and scarring during the healing process [35]. This form of capsular retraction tends to be localised, small and peripheral to the zone of injury, sometimes with adjacent hypodense areas of fibrosis [35]. Examples of procedures causing iatrogenic hepatic trauma include liver biopsy, percutaneous biliary drainage or percutaneous drainage of hepatic abscesses or cysts, as well as partial hepatectomy [35]. In these cases, a history of hepatic interventions should alert the radiologist to the possibility of iatrogenic hepatic trauma as a cause for hepatic capsular retraction.

#### Treated tumours

**Fig. 20:**
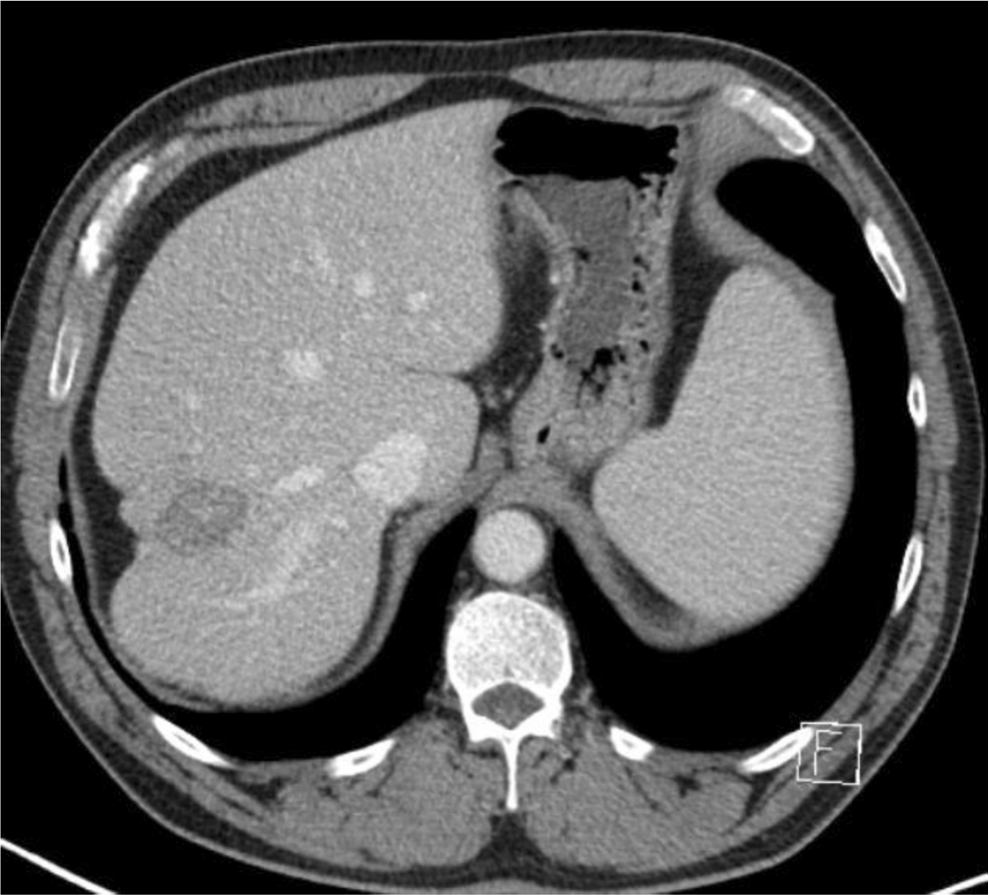
Axial portal venous CT demonstrating capsular retraction in segment 7/8 with a hypo-enhancing ablation zone deep to it

**Fig. 21:**
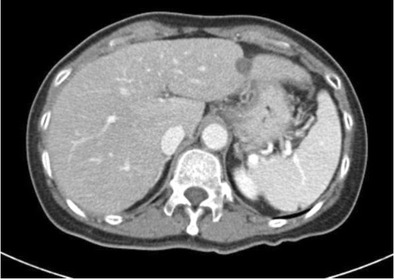
Hypo-enhancing focus with capsular retraction in segment 2, at a site of radiofrequency ablation performed for hepatocellular carcinoma 12 months earlier

Minimally invasive ablation techniques have increasingly important roles in the management of hepatic tumours, particularly in patients who are unfit for surgery. Multiple ablation techniques have been employed in the treatment of liver tumours, including radiofrequency ablation (RFA), cryoablation, high-intensity focused ultrasound (HIFU), ethanol injections and microwave ablation (MWA). These ablative techniques result in coagulative necrosis of the tumour tissue, which eventually involutes and is replaced by fibrous scar tissue, which in turn can result in hepatic capsular retraction [36, 37] (Figs. 20 and 21).

#### Pseudomyxoma peritonei

**Fig. 22:**
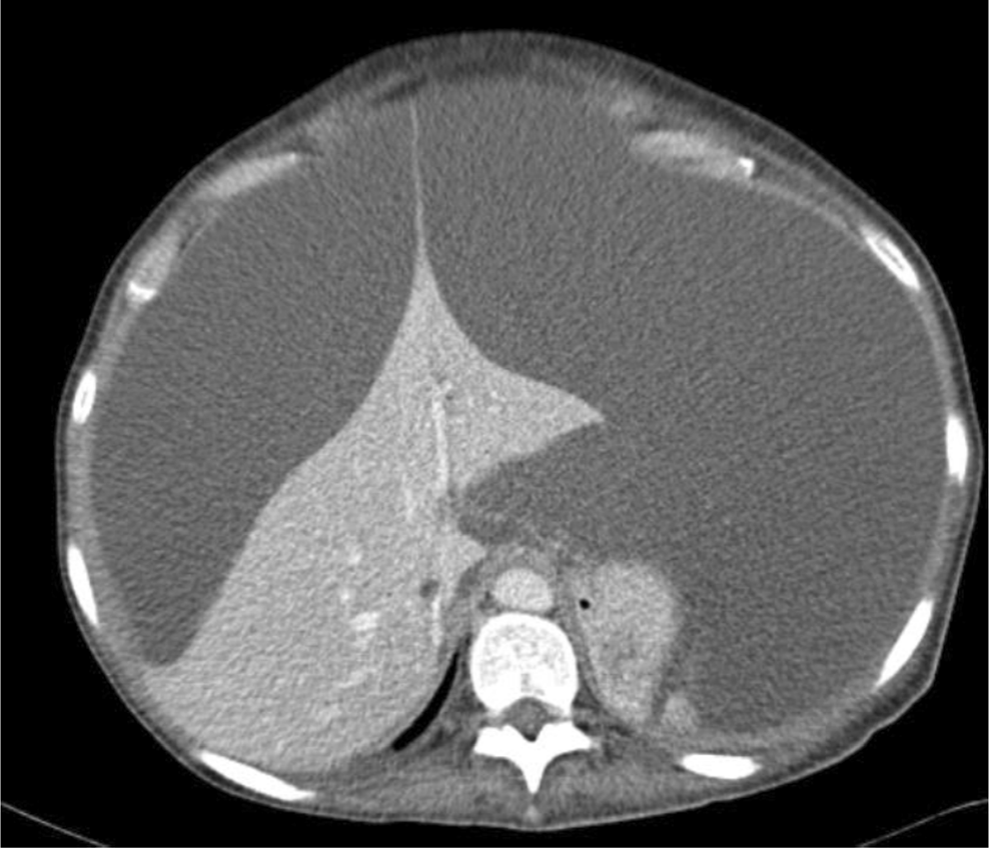
Pseudomyxoma in a 60-year-old woman with ovarian carcinoma. The peritoneal cavity is massively expanded and, under pressure, manifest as compression and displacement of the liver. Note that the stretched paraumbilical ligament still anchors the liver to the anterior abdominal wall

Pseudomyxoma peritonei is the diffuse accumulation of gelatinous ascites due to the rupture of well-differentiated mucinous tumours. The most common cause is ruptured mucinous tumour of the appendix [38]. Mucinous tumours of the colon, rectum and pancreas and urachal tumours have also been shown to cause pseudomyxoma peritonei [39]. There is some controversy regarding pseudomyxoma peritonei due to rupture of tumours of the ovary, and whether these are in fact appendiceal tumour metastases rather than primary ovarian tumours [40, 41]. On CT, pseudomyxoma peritonei appears as low-attenuation, often loculated fluid throughout the peritoneum, omentum and mesentery. Scalloping of visceral surfaces, particularly in the liver, with distortion of the capsule is a very typical feature and allows differentiation from simple ascites. This capsular distortion may mimic hepatic capsular retraction on imaging (Fig. 22). Curvilinear calcification may also be present. On MRI, the gelatinous ascites typically demonstrates low signal on T1 and high signal on T2, and may show some enhancement post-administration of gadolinium contrast.

#### Pseudolipoma of Glisson’s capsule

**Fig. 23:**
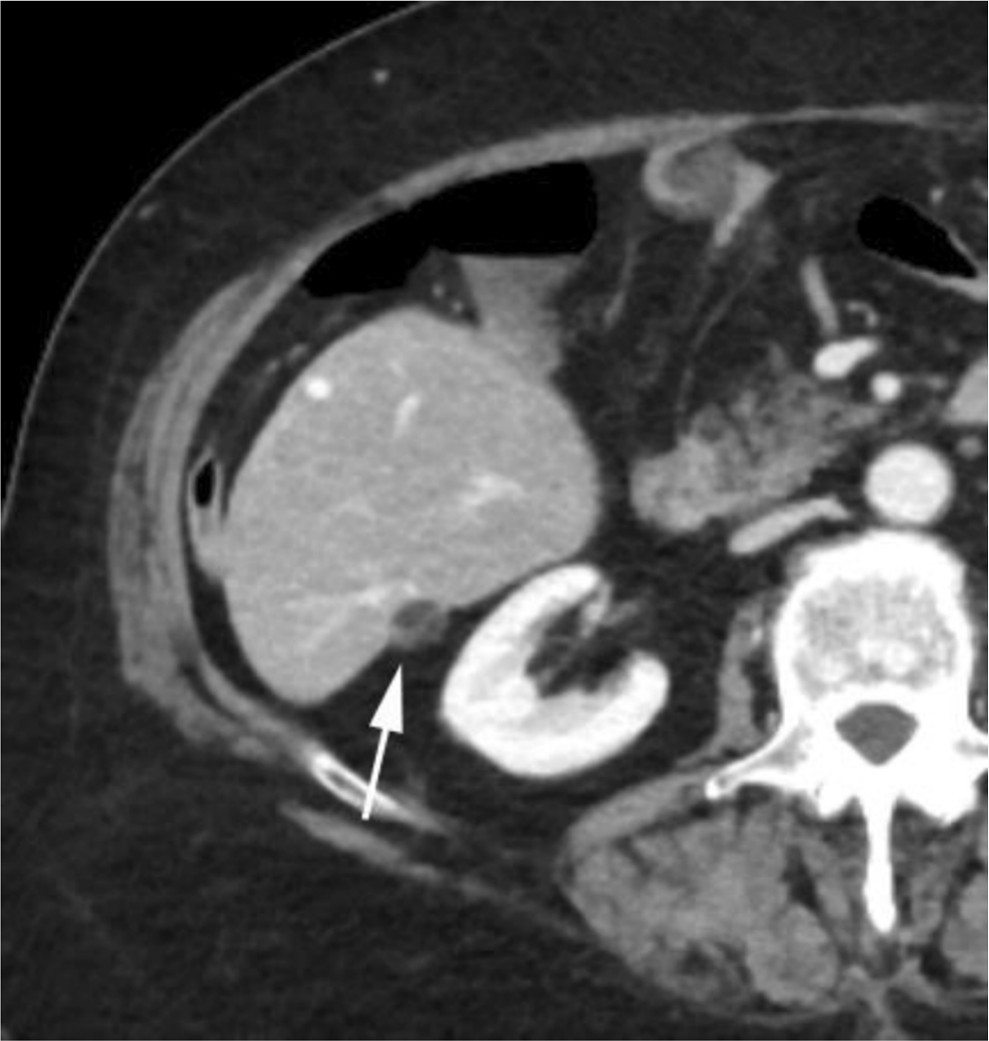
Portal venous phase CT showing a small fatty capsular implant (*arrow*) at the deep aspect of the liver within Morrison’s pouch in keeping with a pseudolipoma of Glisson capsule. It indents the underlying hepatic parenchyma

**Fig. 24:**
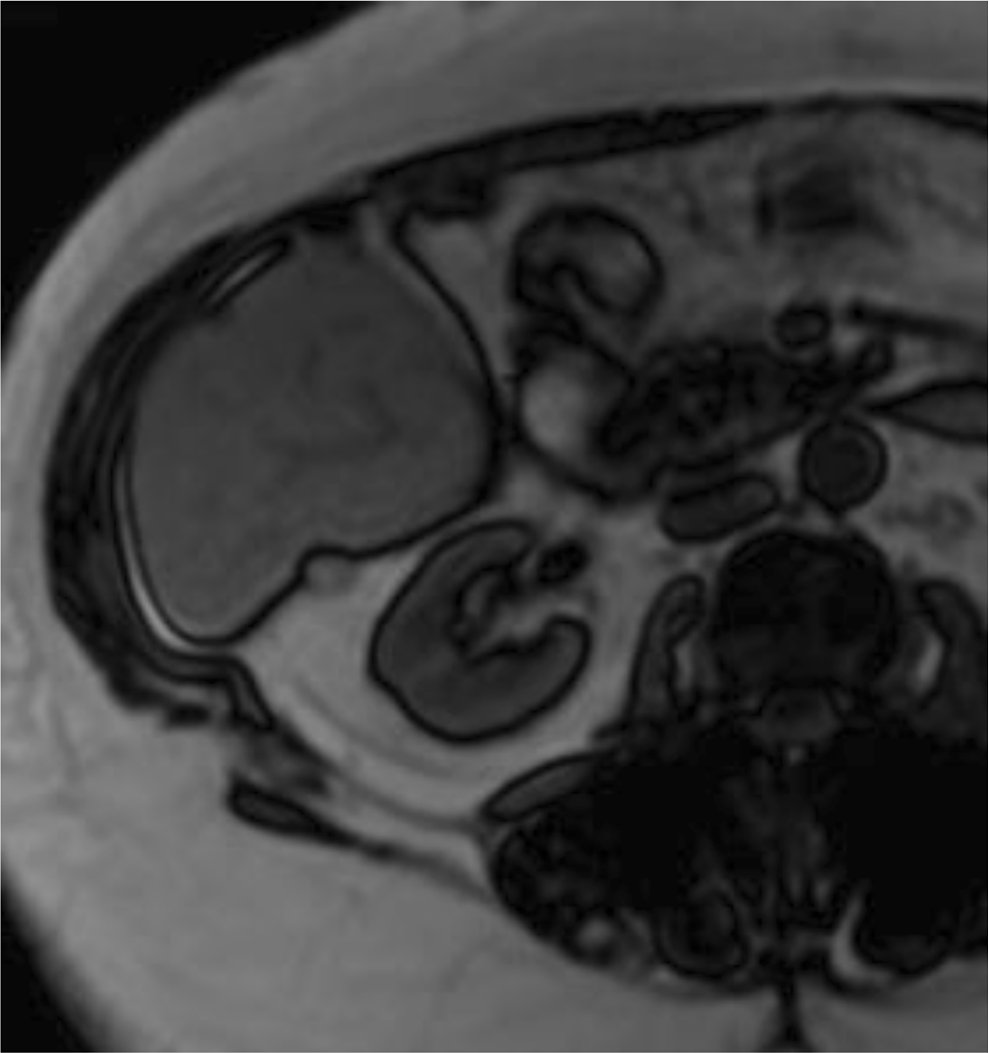
Opposed-phase T1-weighted image demonstrating ‘india ink’ artefact between the pseudolipoma and the liver, but not between the pseudolipoma and adjacent fat in Morrison’s pouch

A rare benign tumour of the liver, pseudolipoma of Glisson’s capsule is an encapsulated lesion containing necrotic fatty tissue, attached to the surface of the hepatic capsule [42]. It is hypothesised to originate from a torted, detached epiploic appendage, which becomes adherent to the hepatic capsule [42]. On CT imaging, this tumour appears as a well-defined nodule of fat attenuation along the hepatic capsule, causing indentation of the underlying hepatic parenchyma [43], which appears similar to hepatic capsular retraction (Fig. 23). On MRI, it demonstrates fat signal intensity [43]. On opposed-phase T1-weighted images, the key feature is the presence of an ‘india ink artefact’ between the pseudolipoma and the liver, but not between the liver and adjacent fat (Fig. 24). It is useful to consider pseudolipoma in the differential diagnosis of hypodense hepatic capsular lesions, as it can prevent unnecessary invasive investigations.

## Conclusion

Hepatic capsular retraction is increasingly encountered and may be due to a diverse spectrum of pathologies. A thorough investigation of the region and correlation with patient history can allow a specific diagnosis to be made.
